# Effects of Aggregate Types on the Stress-Strain Behavior of Fiber Reinforced Polymer (FRP)-Confined Lightweight Concrete

**DOI:** 10.3390/s18103525

**Published:** 2018-10-18

**Authors:** Pengda Li, Lili Sui, Feng Xing, Xiaoxu Huang, Yingwu Zhou, Yanchun Yun

**Affiliations:** 1 Guangdong Provincial Key Laboratory of Durability for Marine Civil Engineering, Shenzhen University, Shenzhen 518060, China; pdli.peter@szu.edu.cn (P.L.); suill@szu.edu.cn (L.S.); xingf@szu.edu.cn (F.X.); xxhruby@szu.edu.cn (X.H.); 2 Baoye Group Company Limited, Shanghai 201109, China; yunyanchun@126.com

**Keywords:** lightweight aggregate concrete, stress-strain behavior, fiber-reinforced polymer (FRP), strain sensing, confinement, aggregate types

## Abstract

The realization of reducing concrete self-weight is mainly to replace ordinary aggregates with lightweight aggregates; such replacement usually comes with some intrinsic disadvantages in concrete, such as high brittleness and lower mechanical properties. However, these shortages can be effectively remedied by external confinement such as fiber reinforced polymer (FRP) jacketing. To accurately predict the stress-strain behavior of lightweight concrete with lateral confinement, it is necessary to properly understand the coupling effects that are caused by diverse aggregates types and confinement level. In this study, FRP-confined lightweight concrete cylinder with varying aggregate types were tested under axial compression. Strain gauges and linear variable displacement transducers were used for monitoring the lateral and axial deformation of specimens during the tests. By sensing the strain and deformation data for the specimens under the tri-axial loads, the results showed that the lateral to axial strain relation is highly related to the aggregate types and confinement level. In addition, when compared with FRP-confined normal weight aggregate concrete, the efficiency of FRP confinement for lightweight concrete is gradually reduced with the increase of external pressure. Replace ordinary fine aggregate by its lightweight counterparts can be significantly improved the deformation capacity of FRP-confined lightweight concrete, meanwhile does not lead to the reduction of compressive strength. Plus, this paper modified a well-established stress-strain model for an FRP-confined lightweight concrete column, involving the effect of aggregate types. More accurate expressions pertaining to the deformation capacity and the stress-strain relation were proposed with reasonable accuracy.

## 1. Introduction

Lightweight concrete is generally defined as concrete made of ordinary Portland cement (OPC), water, river sand (or lightweight sand), and lightweight coarse aggregates, and its density is typically below to 1950 kg/m^3^ [1]. Consider the growing demand, including high-rise buildings, large-span concrete structures, and floating structures, lightweight concrete that is made by diverse types of aggregate has been widely studied and successfully developed and applied over the past two decades [1,2,3,4,5,6]. Lightweight concrete offers several advantages, such as saving dead loads for foundations, high strength/weight ratio, and service as ideal filled materials for sandwich structures. Thus, lightweight concrete has many potential applications in the construction industry. Nevertheless, some drawbacks in lightweight concrete’s natural mechanical properties have limited applications, especially as load-bearing structural members [6]. At the same mixing ratio and compressive strength, the brittleness of lightweight concrete is much higher than normal concrete (NC). Plus, the deformation capacity of lightweight concrete is also poor when compared with NC [7].

Fiber-reinforced polymer (FRP) composites have become a favorite material of professionals in both engineering and construction due to its advantages. Major ones include lightweight, high strength, and construction convenience [8,9,10,11,12,13]. FRP composites provide excellent corrosion resistance, which keeps costs down and increases the service life of structural materials. [14,15,16,17,18,19,20,21,22,23]. FRP jacketing of concrete has been widely accepted for retrofitting or repairing concrete members, whereby the strength capacity and ductility of concrete are efficiently enhanced. Numerous related studies have been demonstrated in the past several decades [2,14,24,25,26,27,28,29,30,31,32,33,34,35,36]. The circumferential confinement of FRPs restrains the transverse expansion of concrete; thus, the strength and ductility of FRP-confined concrete are notably enhanced when the concrete is subjected to a triaxial compressive load [37,38,39,40]. Therefore, it can be inferred that the problem of high brittleness and the poor ductility of lightweight aggregate concrete can be effectively solved by using FRP confinement. Thus, FRP application can make it an effective method to reduce self-weight in structural design. The advantage of composite structure is that they can fully use the characteristics of the multi-materials [41,42,43]. However, it also brings more complicated damage mechanism or confinement mechanism of the composite materials. With the developing of sensor or monitoring technology, some smart sensors have been successfully used inside the concrete composite materials to observe the inner damage condition [44,45,46,47,48,49,50]. To understand the damage mechanism of FRP-confined lightweight concrete. It is also necessary to monitor the dilation behavior that is caused by different aggregate types.

Through literature review, the corresponding author Zhou [2] first conducted experiments to test the stress-strain behavior of carbon FRP (CFRP)-confined lightweight aggregate concrete (hereafter referred to as LWAC), where only lightweight coarse aggregates were adopted for reducing self-weight. Zhou et al.’s study [2] revealed that the ultimate strength and ductility capacity of LWAC could be considerably enhanced by jacketing CFRP. It was also found that FRP confinement is more effective for LWAC cylinders than NC regarding both strength and ductility enhancement. Aiming at further reducing the weight of concrete, this study thus proposes a new type of FRP-confined LWAC that fully uses lightweight aggregates as the core materials based on LWAC, where fine lightweight aggregates further instead of ordinary sand was adopted—notably, ceramic sand. Hereafter, this type of lightweight concrete will be referred to as full lightweight aggregate concrete (FLWAC). In this work, an experimental investigation of the stress-strain behavior of FRP-confined FLWAC was conducted. Test results were compared and analyzed with test data from the previous study on FRP-confined LWAC [2], and the effect of the lightweight aggregate material (both coarse and fine aggregate) and the amount of FRP confining pressure regarding its mechanical properties, in terms of failure modes, ultimate condition, and strain-stress behavior of the FRP-confined FLWAC was determined. Then, an improved stress-strain model was proposed for the FRP-confined lightweight concrete, and this model is capable of analyzing diverse types of lightweight aggregates.

## 2. Experimental Database

### 2.1. Existing Test Data for FRP-Confined Lightweight Aggregate Concrete

In this study, the results of a new experiment were analyzed together with the existing test data from Zhou et al. [2] to study the effects of lightweight aggregate (both coarse and fine aggregate) on the mechanical properties of FRP-confined lightweight concrete. Details of these studies are presented in the following subsections. Zhou et al.’s test data included a total of 12 CFRP-confined lightweight concrete cylinders in which normal coarse aggregates were replaced solely by lightweight coarse aggregates (Grade 800 and Grade 600 crushed shale ceramsite manufactured by Yichang Baozhu Ceramics Development Co., Ltd., Yichang, Hubei, China) [2]. All of the concrete cylinders in this series of tests were 150 mm in diameter and 300 mm in height. The concrete strengths were determined by an axial compression test, which is 21.2 and 38.8 MPa for Grade 600 and Grade 800 crushed shale ceramsite, respectively. One or three layers of CFRP were wrapped around the concrete cylinder in a wet lay-up method, and all specimens underwent axial compression until failure. The details of the experimental results are shown in Table 1.

### 2.2. The New Test

#### 2.2.1. Materials Properties

**Figure 1:**
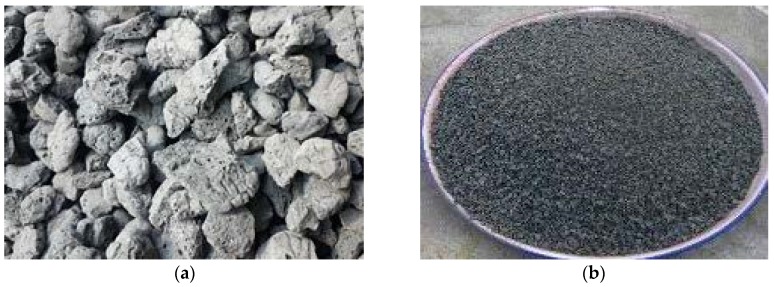
Appearance of the lightweight coarse and fine aggregates: (**a**) Crushed shale ceramsite; (**b**) Ceramic sand.

In this study, lightweight concrete was prepared using ordinary Portland cement (OPC), water, lightweight fine aggregates (LFAs), lightweight coarse aggregates (LCAs), and silica fume. Table 2 shows the mix proportion in which Grade 800 crushed shale ceramsite (Figure 1a), was used as the coarse aggregate and its particle sizes range from 5 to 20 mm; the main properties of LCAs are given in Table 3; Continuous gradation ceramic sand (Figure 1b) with a diameter less than 3 mm was adopted as the fine aggregate. The gradation features for LCAs and LFAs are shown in Table 4 and Table 5, respectively. The dry density of FLWAC was only 1581 kg/m^3^, which is much lower than NC’s 2400 kg/m^3^ and LWAC’s 1776 kg/m^3^, since lightweight fine aggregates were used. To obtain the mechanical properties of FLWAC, three unconfined cylinders as control specimens were tested under compression. Results show that the compressive strength of the FLWAC is 39.8 MPa, but its elastic modulus is only 22.0 GPa, which is lower than that of LWAC’s 26.8 GPa reported by [2], even the compressive strength of LWAC in C2 group (38.9 MPa) is close to FLWAC. After 28 days, the FLWAC was jacketed by CFRP laminate (0.167 mm in thickness) in a hoop direction using the wet lay-up manner with an overlap of 150 mm. The adhesive was a two-part Lica^®^-100 impregnated epoxy resin. Through the coupon test, the CFRP material properties were obtained, which are ultimate strain (1.31%), ultimate tensile strength (3770 MPa), and elastic modulus (287 GPa), respectively. The coupon testing was carried out according to ASTM D3039 [51], and each coupon test specimen is a one-layer CFRP sheet.

#### 2.2.2. Specimens Design

A total of 13 FLWAC cylinders 150 mm wide × 300 mm high were prepared. To achieve an even stress distribution as the compressive load was applied, all specimens were topped by gypsum plaster. The columns were categorized according to the FRP layers and the concrete grade. Table 1 shows the details of each specimen based on their definition in the experiments. The specimen terms with the letter C plus a number representing FLWAC strength is followed by the letter F plus the FRP ply number. The last number denotes the number of replicated specimens. For example, C40F1-3 denotes a No. 3 specimen with a grade C40 FLWAC column wrapped in one-ply CFRP.

#### 2.2.3. Experimental Setup and Instrumentation

**Figure 2:**
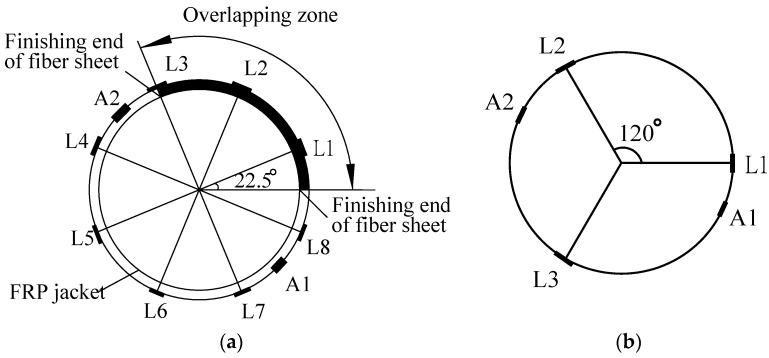
Distribution of strain gauges: (**a**) carbon Fiber-reinforced polymer (CFRP)-wrapped FLWAC; and, (**b**) Plain FLWAC.

**Figure 3:**
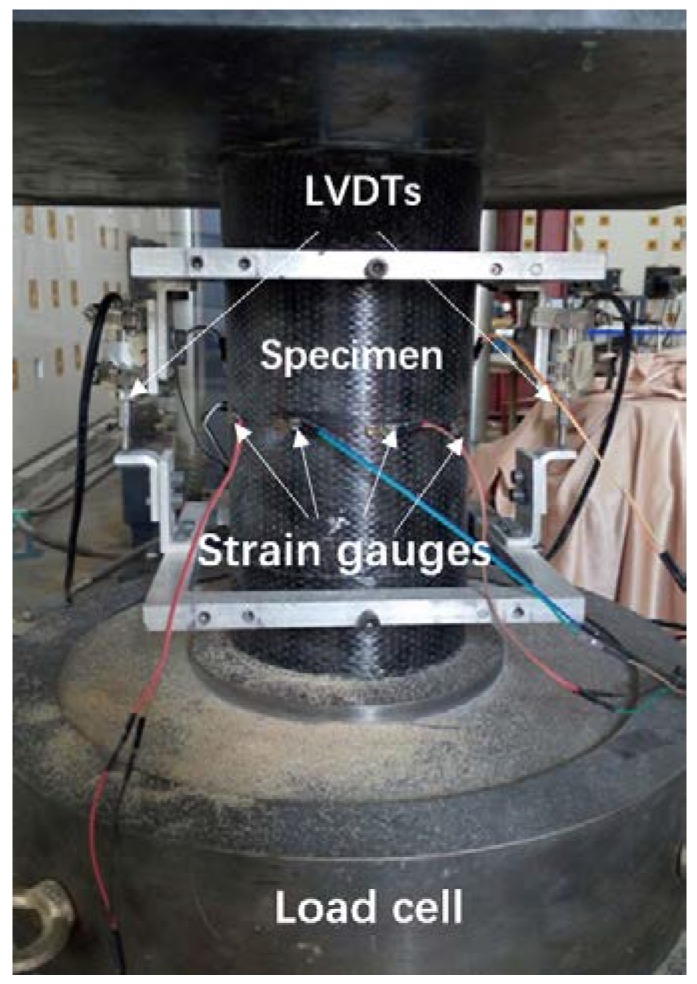
Test setup.

In this experiment, ten 10-mm strain gauges were mounted on the mid-height of each FRP-confined specimen surface, in which two for axial strain measurement and eight for hoop strain measurement. Three of the eight hoop gauges were placed within the overlapping zone. For FLWAC without FRP confinement, three strain gauges were used to measure the hoop strain. Figure 2 demonstrates the distribution of the strain gauges for CFRP-wrapped FLWAC and plain FLWAC, where A denotes the axial strain gauges and L represents the lateral strain gauges (hoop strain gauges). Also, two LVDTs were utilized to obtain the axial displacement of the 185 mm over the middle height for each specimen as shown in Figure 3. All the tests were conducted using a compression machine with a maximum load capacity of 3000 kN. The cylinders were first loaded by force control with a loading rate of 1 kN/s to the initial elastic portion up to 70% of the FLWAC strength, and the loading mode was then converted to displacement control with a rate of 0.3 mm/min until the failure of specimen.

## 3. Test Results

### 3.1. Failure Observations

**Figure 4:**
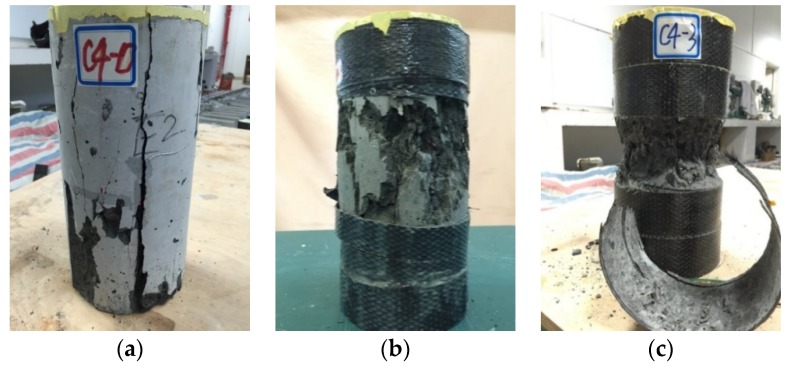
Failure modes of representative fiber-reinforced polymer (FRP)-confined concrete cylinders: (**a**) Unconfined; (**b**) 1-ply CFRP confined; and, (**c**) three-ply CFRP confined.

As revealed in Figure 4, these test specimens finally failed with three typical failure modes. Longitudinal cracks (from splitting) were observed for the specimen without CFRP confinement. The major damage to the concrete cylinder was captured along the vertical cracks and it covered the whole section of the FLWAC column from top to bottom. For CFRP-confined FLWAC, as shown in Figure 4b,c, specimens failed due to tensile CFRP rupture due to the expansion of their inner concrete core. Furthermore, the crushed concrete can be seen behind the fiber rupture zone. Notably, with the increase of CFRP plies, the CFRP rupture region was more concentrated, and the concrete was almost crushed into powder for the three-ply CFRP confinement. The observed variations in the failure mode patterns demonstrated that the damage condition of the FLWAC became worse with the increased lateral confinement, which altered the FLWAC crack patterns from vertical macrocracks to localized microcracks. Compared with NC and LWAC, as reported in existing literature, this localization phenomenon is more pronounced for FRP-confined FLWAC. It is attributed to the poriferous characteristics of lightweight fine and coarse aggregates, leading to their increased softening, making them easy to be crushed.

### 3.2. Experimental Stress-Strain Curves of FRP-Confined FLWAC

**Figure 5:**
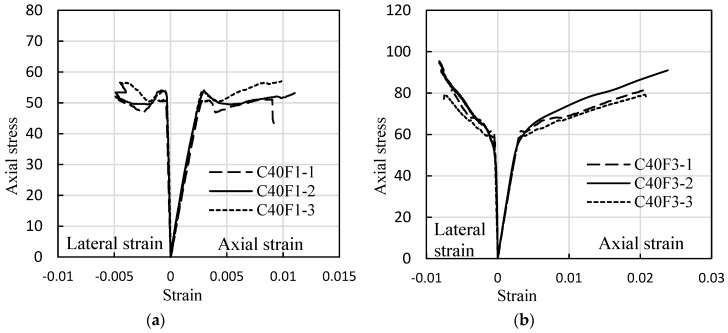
Stress-strain curves of FRP-confined FLWAC. (**a**) one-ply FRP-confined FLWAC; (**b**) three-ply FRP-confined FLWAC.

Figure 5 shows the axial compressive stress-strain behavior of all the FRP-confined FLWAC cylinders. Much like the stress-strain curves of FRP confined NC [29,52,53,54], the curves for FRP-confined FLWAC also display a monotonically ascending bi-linearity, and the stiffness rapidly decreases after the transition zone. Previous researchers have intensively studied the stress determined the transition zone for FRP-confined NC, and it is concluded that the stress is around the strength of the unconfined concrete [17]. For FRP confined FLWAC, however, the stress is highly related to the amount of wrapped CFRP. Figure 5 shows that, for the unconfined concrete strength of 39.8 MPa, the transition stress was enhanced to 50 MPa for one-ply FRP confinement and 60 MPa for a three-ply FRP confinement. The observed variations are attributed to the brittleness of the lightweight concrete, which became more limited with the increased lateral confinement. In other words, the confinement postponed sudden failure.

**Figure 6:**
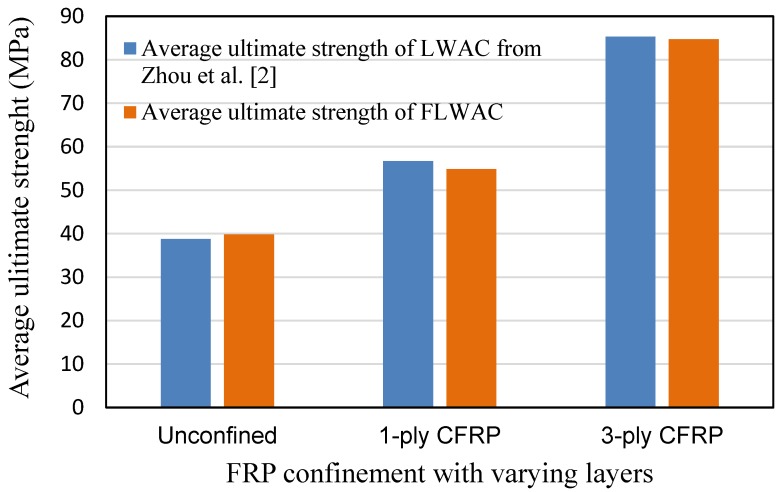
The load bearing capacity of the CFRP-confined FLWAC columns and LWAC.

Figure 6 compares the unconfined strength of the LWAC and FLWAC columns with their CFRP-confined counterparts with particular attention to varying CFRP ply. The strength of both LWAC and FLWAC columns was observed to be dramatically enhanced by the same rate related to the amount of CFRP confinement (number of layers) and their unconfined strength. These comparisons also show that using fine lightweight aggregates instead of ordinary fine aggregates (river sand) has little effect on their ultimate strength.

## 4. Discussions

### 4.1. Hoop Strain Distribution and Effective Hoop Strain Factor

**Figure 7:**
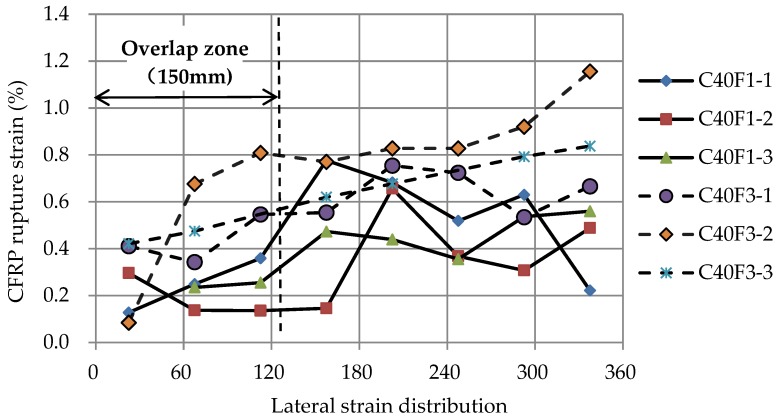
Lateral strain distribution.

The maximum confinement pressure provided by FRP is defined by the tensile strength at the FRP rupture moment. However, the testing rupture strain value is usually less than the coupon test value [17,27,55]. Therefore, the effective hoop rupture strain at the FRP rupture is a vital parameter to predict the axial stress-strain behavior accurately. Equation (1) is the expression of the effective hoop confinement stress *f_le_*,

(1) $$f_{le}=\frac{2E_{f}t_{f}\varepsilon_{h.rup}}{D}\;$$

where *D* is the cross-sectional diameter of the specimen; *t_f_* and *E_f_* are the thickness and stiffness of the CFRP sheet, respectively; and, *ε_h_*_,*rup*_ is the effective rupture strain of FRP, which is given by:

(2) $$\varepsilon_{h,rup}=k_{\varepsilon }\varepsilon_{f}\;$$

where *ε_f_* represents the CFRP tensile rupture strain obtained from coupon tests and *k_ɛ_* is the effective hoop strain factor, which can describe the effective utilization ratio of the CFRP material as it ruptures. Previous reports have indicated that this factor is related to the hoop strain distribution [27,55]. As shown in Figure 7, the distribution of the hoop rupture strain for CFRP-confined FLWAC cylinders at their failure point was non-homogeneous. For all specimens, the maximum lateral strain appeared outside the overlapping zone, which replicates the test results from other types of FRP-confined concrete [2,25,29]. Notably, the rupture strain of concrete wrapping with three-ply CFRP demonstrated a relative higher average value and a more uniform cracking pattern compared with that of concrete wrapping with 1-ply CFRP. According to the observed failure modes shown in Section 3.1, the damage pattern is more concentrated and uniform in specimens with three-ply CFRP confinement, which causes a more even distribution of hoop strains resulting in a larger average rupture strain for specimens with a thicker FRP confinement than that of specimens with a smaller amount of FRP. The calculated effective hoop strain factor is also shown in Table 1. However, existing data for FRP-confined FLWAC is not sufficient to propose a new effective hoop strain factor. In the present paper, after combining this research’s results with test results from Zhou et al. [2] for FRP-confined LWAC, the average value of *k_ε_* = 0.53 was used for the analysis before the quantitative study of the effective hoop strain factor of the FRP-confined lightweight concrete was available.

### 4.2. Lateral to Axial Strain Relationship

**Figure 8:**
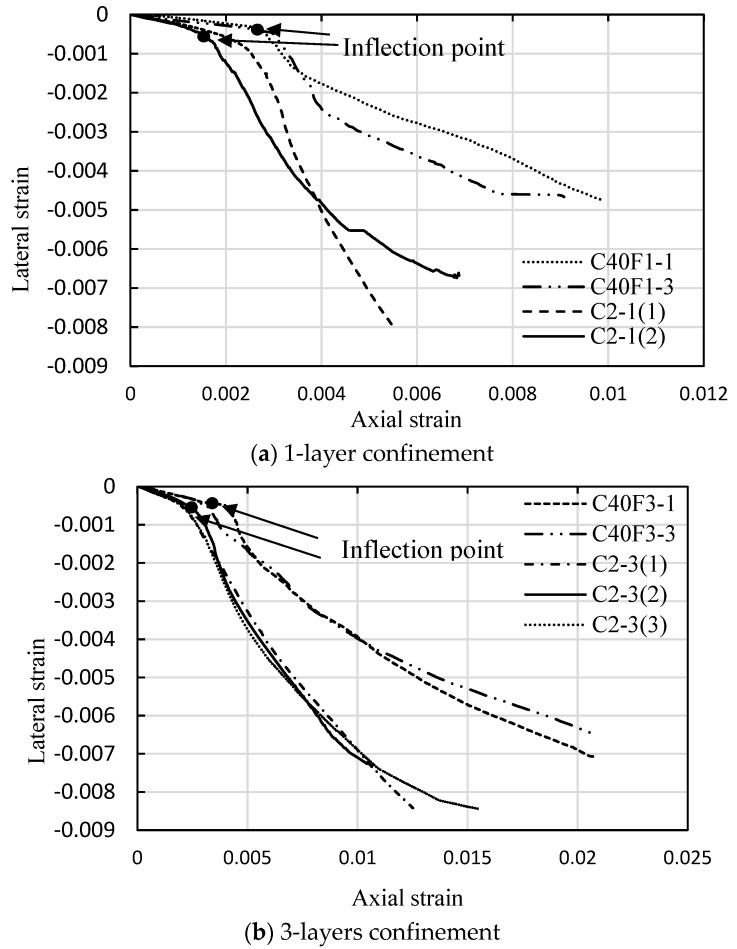
Comparison of lateral to axial strain curves for FRP-confined Lightweight concrete with similar unconfined concrete strength and different aggregate type.

Unconfined concrete starts rapidly volumetric expansion as its axial compression exceeds to 90% of the peak strength, and the fine aggregate effects on the post-peak behavior are not apparent due to the rapid drop in stress. However, this lateral dilatation can be effectively restrained by external FRP confinement, and this increasing expansion of concrete passively generates a continuously increasing lateral confining pressure that is provided by the FRP jacket. The dilation properties of FRP-confined concrete are reflected by its lateral to axial strain relationship. Figure 8 shows the comparison of lateral to axial strain curves for FRP-confined FLWAC in this study and LWAC from Zhou et al. [2], where the concrete strength of all specimens is almost same (39.8 for FLWAC and 38.8 MPa for LWAC). These figures show the lateral-axial strain curves possess a similar pattern, which shows bilinear behavior separating them from the transition zones where concrete expansion accelerates. After the inflation points are formed, the slope of the second part of curves shows significantly different, and an even steeper slope can be found for LWAC specimens or both one- and three-layer FRP confinements. It means that FRP-confined FLWAC shows a larger axial strain than that of FRP-confined LWAC when their lateral strains are same. This could be attributed to the usage of fine lightweight aggregates in FLWAC, resulting in more deformation in the axial direction. In addition, the inflection points for FRP-confined FLWAC is consistently delayed compared to the predicted curve for LWAC, as shown in Figure 8. This is also an evidence that the external FRP was activated later for FRP-confined FLWAC due to “softer” in axial deformation because of containing the fine lightweight aggregate that delays its dilation behavior. Therefore, aggregate type plays a major role on the lateral to axial strain relation for FRP-confined concrete. This test results and conclusion is first proposed and is important for understanding the dilation behavior of FRP confined concrete. Previous study indicated that the dilation behavior of FRP confined concrete is only governed by the concrete grade and lateral confinement pressure [54]. However, according to this study, the dilation behavior of FRP-confined lightweight concrete is also highly related to the fine aggregate properties. Moreover, the different fine aggregates types could further affect the ultimate condition of FRP-confined lightweight concrete, the discussion is given in the following sections.

### 4.3. Ultimate Strength Model

The majority of strength models in literature for confined concrete take the following mathematical form:

(3) $$\frac{f_{cc}}{f_{co}}=1+k_{1}(\frac{f_{l}}{f_{co}}{)}^{k_{2}}\;$$

where *f_co_* and *f_cc_* provide the peak strength of the unconfined and confined concrete, respectively. *f_l_* is the lateral confining pressure (or effective confining pressure *f_le_*) that can be obtained from Equation (1). *k*_1_ is the coefficient representing confinement effectiveness. This expression originally was developed by Richard et al. [56] for spiral steel wire confined concrete with a value of 4.1 for *k*_1_. A linear relation was found between *f_cc_*/*f_co_* and *f_l_*/*f_co_*; thus, *k*_2_ is equal to 1. However, earlier studies [19,20,34,57,58] revealed that the existing strength models are suitable for steel reinforced concrete could not be applicable to FRP-confined concrete, and the coefficient *k*_1_ is not a constant dependent on a different database and parameters, such as aspect ratio, corner radius, concrete strength, etc. Recent studies [2] indicate that the *k*_1_ is also related to the type of aggregate because the bond behavior within the interfacial transition zone between the aggregate and cement matrix differs significantly with any variation of aggregate type. Furthermore, the interfacial bond behavior dislikes a linear increase relation between the confinement ratio *f_l_*/*f_co_* and the ultimate strength ratio *f_cc_*/*f_co_* for NC; thus, the rate of strength gain *f_cc_*/*f_co_* for FRP-confined LWAC gradually decreases with the rising confinement ratio when *f_l_*/*f_co_* > 0.4, resulting in a nonlinear trend line, as shown in Figure 8. After a thorough quantitative assessment of the performance of a compressive strength model between FRP-confined NC and FRP-confined LWAC, Zhou et al. [2] proposed a model for LWAC, expressed as:

(4) $$\frac{f_{cc}}{f_{co}}=1+2.11(\frac{f_{le}}{f_{co}}{)}^{0.65}\;$$

**Figure 9:**
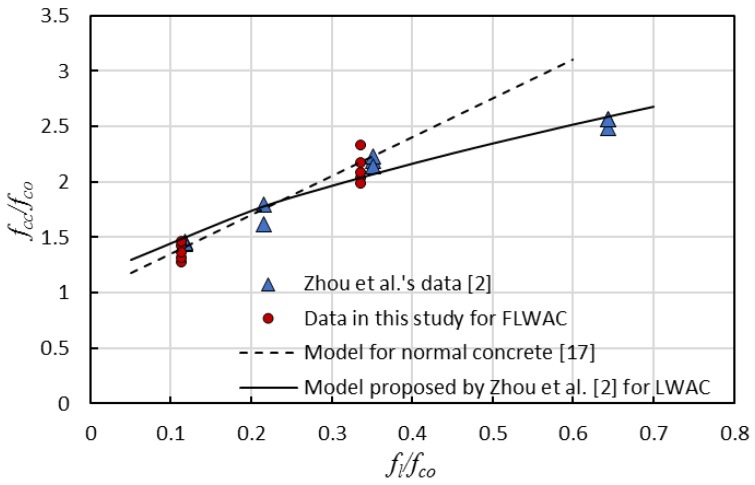
Relation between the ultimate strength ratio and the confinement ratio.

**Figure 10:**
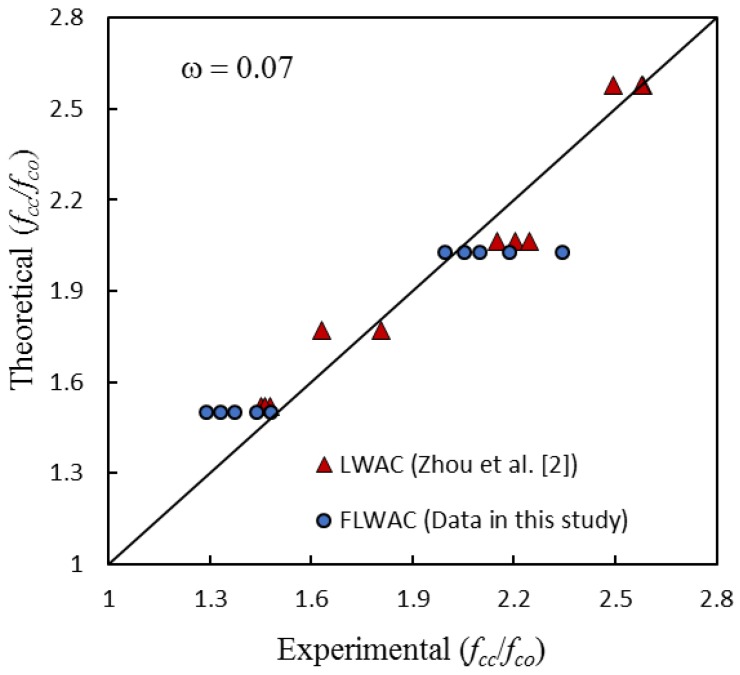
Performance of Equation (4).

As shown in Figure 6 and Figure 9, the enhanced ultimate compressive strengths for FRP-confined FLWAC are almost identical to LWAC; thus, Zhou et al.’s strength model (Equation (4)) is used in this paper for FRP-confined FLWAC. The performance of the proposed ultimate strength model is demonstrated in Figure 10. The error index was evaluated in terms of *ω*, where *Expe.* and *Theo.* represent the experimental and theoretical value, respectively, as given by:

(5) $$\omega =\frac{\sum \left|Expe.-Theo.\right|}{\sum \left|Expe.\right|}\;$$

As shown in Figure 10, the model proposed by Zhou et al. [2] can predict the ultimate strength for both FRP-confined FLWAC and LWAC with good accuracy.

### 4.4. Ultimate Strain Model

Currently, it is generally assumed that the ultimate strain (*ε_cu_*) of FRP-confined NC is related to the amount of FRP confinement and to unconfined concrete strength aspect parameters. Teng et al. [17] improved the understanding of the ultimate strain and first introduced two key parameters, which possessed explicit physical meanings: the rupture strain ratio *ρ_ε_*, and the FRP rigidity ratio *ρ_k_*, to propose the ultimate strain model. These two parameters are given as:

(6) $$\rho_{k}^{}=\frac{2E_{f}t_{f}}{\left(f_{co}/\varepsilon_{co}\right)D}\;$$

(7) $$\rho_{\varepsilon }^{}=\frac{\varepsilon_{h,rup}}{\varepsilon_{co}}\;$$

where the *ε_co_* is the corresponding strain at peak stress for unconfined concrete. According to these parameters suggested by Teng et al. [17], Zhou et al. [2] developed Equation (8) to estimate the axial deformation of FRP-confined LWAC. Where a parameter *λ* quantified the effect of bond strength regarding the interface between the aggregate and cement matrix on the ultimate strain, and the value of *λ* was defined by the aggregate type. For the lightweight coarse aggregate material, *λ* = 1.45.

(8) $$\frac{\varepsilon_{cu}}{\varepsilon_{co}}=1.5+5.24\rho_{k}^{\lambda }\rho_{\varepsilon }^{2.63}\;$$

**Figure 11:**
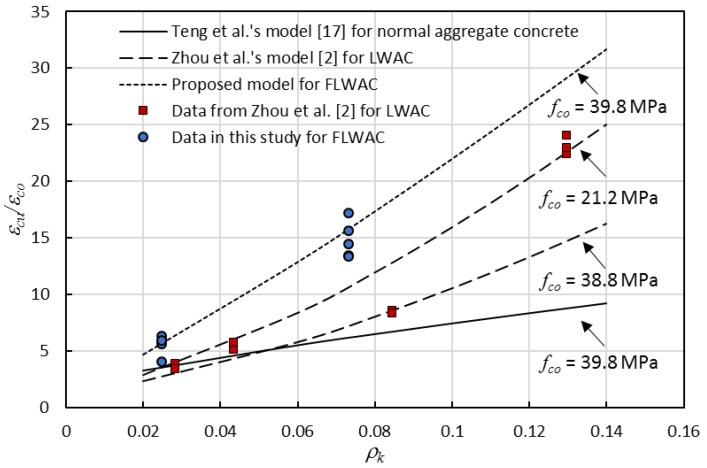
Ultimate strain vs. confinement stiffness ratio.

**Figure 12:**
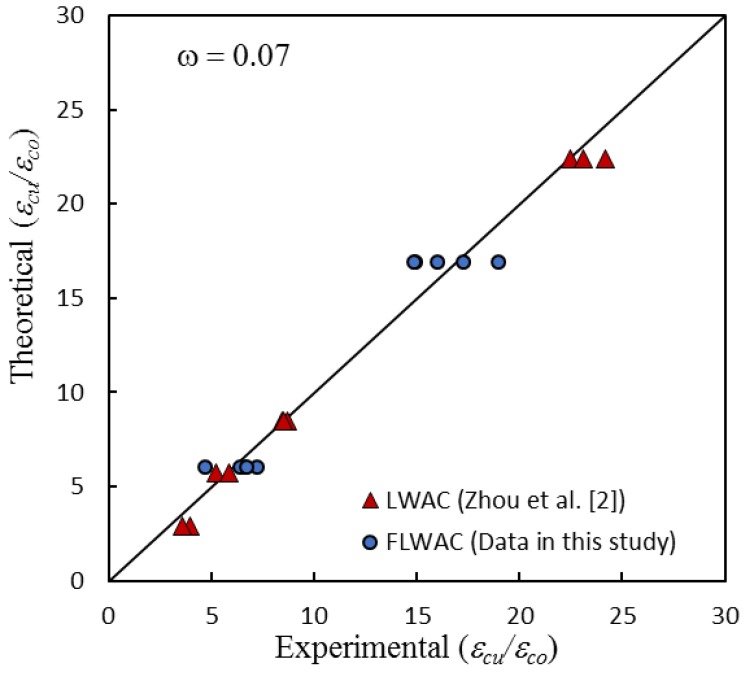
Performance of proposed model against the present test results.

Figure 10 shows the comparison of the ultimate strain of specimens with different aggregate types. Test data in Figure 11 includes the new test results in Table 1, the model line that was derived by Zhou et al. [2] for FRP-confined LWAC, and the model line generated by Lam and Teng [17] for FRP-confined NC. Four different curves with respect to the three different types of FRP confined concrete were plotted together. Figure 11 also shows that the ultimate strain is highly dependent on the FRP rigidity ratio *ρ_k_* and unconfined concrete strength *f_co_*. For the LWAC cylinder, the ultimate strain of a specimen featuring lower concrete strength is generally higher than that of specimens with higher strength concrete, and the impact is more pronounced with the rise in the confinement stiffness ratio. Furthermore, the results also indicate that the ultimate strain is highly dependent on the type of aggregate. For a similar unconfined concrete strength, the ultimate strain ratio (*ε_cu_*/*ε_co_*) for FRP-confined FLWAC is more significant when compared with FRP-confined LWAC and NC. Furthermore, this effect also became more pronounced when the *ρ_k_* increased, which confirms that the increase in ductility due to the FRP confinement is notably larger for FLWAC as compared with NC and LWAC. Thus, the ultimate strain model for LWAC is not applicable to FRP-confined FLWAC. Through a regression analysis of the test data (Figure 10) while using Equation (8), the coefficient *λ* was determined as 1.15, and the model was modified to Equation (9). Figure 12 demonstrates the performance of the proposed model (Equation (9)), where *ω* = 0.07.

(9) $$\frac{\varepsilon_{cu}}{\varepsilon_{co}}=1.5+5.24\rho_{k}^{1.15}\rho_{\varepsilon }^{2.63}\;$$

## 5. Stress-Strain Model for FRP-Wrapped FLWAC

### 5.1. Stress-Strain Relation

**Figure 13:**
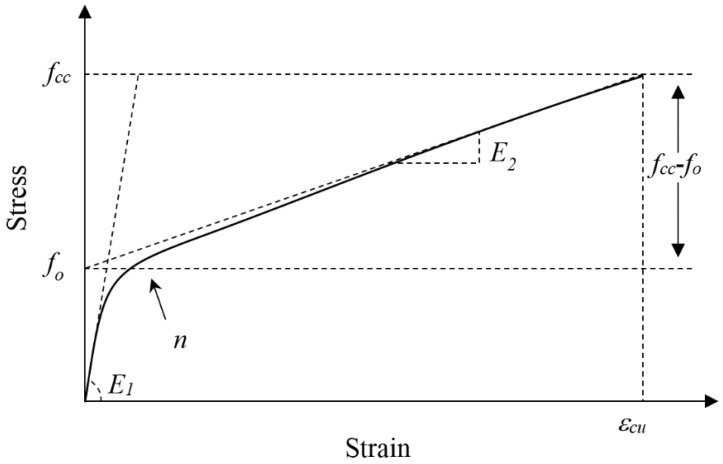
Typical stress-strain curve for FRP confined FLWC.

As shown in Figure 5, the framework of the stress-strain curve for FRP-confined FLWAC is much like the stress-strain relation of FRP-confined LWAC or NC (Figure 13), which also shows a bilinear curve with a softening transition zone: The first linear prat follows a linear trend with a slope that matches the elastic modulus of the unconfined FLWAC; then, the second linear part follows a lower stiffness than the previous one after entering into a nonlinear transition zone. Zhou and Wu’s [59] four-parameter expression (*E*_1_, *f_o_*, *E*_2_, and *n*) was adopted to fit the stress-strain curves of the FRP-confined FLWAC, and this expression is monotonic, continuous, and capable of integration and derivation. This expression [59] has been successfully applied to describe the stress-strain behavior of FRP-confined NC subject to different conditions regarding concrete columns with different cross-sectional shapes, load-induced damaged concrete, environmentally impacted concrete, and eccentric loads [14,20,42,50]. The FRP-confined LWAC also can be described by the stress-strain model, which is given as:

(10) $$f_{c}=\left[\left(n-1\right)f_{o}e^{-\left(E_{1}\varepsilon_{c}/nf_{o}\right)}+f_{o}+E_{2}\varepsilon_{c}\right]\left(1-e^{-\left(E_{1}\varepsilon_{c}/nf_{o}\right)}\right)\;$$

where *E*_1_ and *E*_2_ are the first and second slopes concerning the stress-strain curve. *f_o_* intercepts the second straight line segment with the *y*-axis (see Figure 13), and *n* is the shape factor that governs the nonlinearity degrees of the transition zone.

### 5.2. Determination of Parameters E_1_, f_0_, E_2,_ and n

Both unconfined and confined FLWAC specimens demonstrate a similar slope at the first branch of the stress-strain curve. This is because, before a notable transverse expansion occurs that is defined as the stress-strain curve before transition zone, FRP remains a passively confining mechanism and has not been effectively activated. Hence, the elastic modulus (*E_c_*) of plain FLWAC was directly used to create the first slope of the first curve segment:

(11) $$E_{1}=E_{c}\;$$

As discussed in Section 3.2, the replacement of fine aggregate by lightweight aggregates has little effect on strength related parameters. Hence, in this study, the *f_o_* model that was developed by Zhou et al. [2] for FRP-confined LWAC can be directly adopted.

(12) $$f_{o}=f_{co}+0.8f_{le}+10.7\;$$

**Figure 14:**
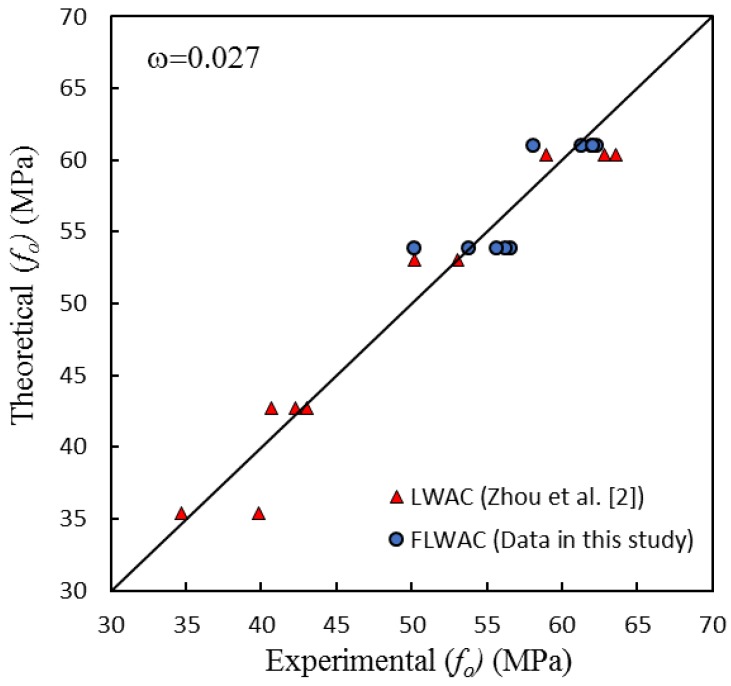
Performance of Equation (12).

Figure 14 shows the performance of model *f_o_* compared with the test results for both the FRP-confined FLWAC and LWAC, which gives an error index where *ω* = 0.027. The good performance validated the previous conclusion, stating that using fine aggregate does not affect the strength related parameter *f_o_*. Moreover, the proposed FRP-confined LWAC model is also capable of predicting this parameter for the FRP-confined FLWAC.

*E*_2_ can be directly obtained by the geometric relation of the stress-strain curve. As shown in Figure 13, the *E*_2_ is the gradient of the line between points (*ε_cu_*, *f_cc_*) and (0, *f_o_*)):

(13) $$E_{2}=\frac{f_{cc}-f_{o}}{\varepsilon_{cu}}\;$$

where *f_cc_*, *f_o_*, and *ε_cu_* can be calculated using Equations (4), (9) and (12), respectively.

Previous studies have concluded that parameter *n* is not sensitive with variables, such as concrete strength and confinement [2,14,60]. Parameter *n* is only related to the nonlinearity degree of the transition zone, and the value of *n* is normally between 0 and 1. The value of 0.5 was suggested by Zhou et al. [2] for FRP confined LWAC. Based on this new data for FRP-confined FLWC and Zhou et al.’s test results for LWAC [2], the regression result of the *n* value ranges from 0.46 to 0.54. The *n* value is little dependent on the aggregate type and number of FRP layers. For the sake of simplification, the mean value of 0.5 is used to define parameter *n*.

## 6. Evaluation of the Stress-Strain Model

**Figure 15:**
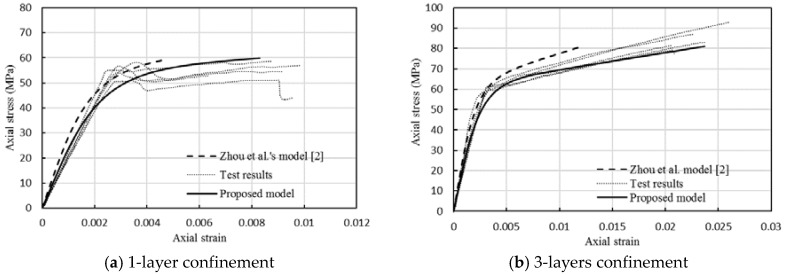
Comparison of experimental stress-strain curves with predictions of the proposed model.

The stress-strain curve of the FRP-confined FLWAC can be generated using Equations (4)–(13). Curves derived using the model proposed for FRP-confined FLWAC and the model of FRP-confined LWAC from [2] are plotted together with test data, as revealed in Figure 15. Thus, Figure 15 indicates the differences in the stress-strain relationship between FRP-confined FLWAC and LWAC. The comparison shows that the stress-strain model developed by Zhou et al. [2] for FRP-confined LWAC does not apply to the FRP-confined FLWAC due to the underestimated ultimate strain. This effect not only applies to the determination of ultimate strain, but also relates to the stiffness of the second curve segment model because the ultimate strain was used to calculate *E*_2_. However, the model proposed in this work successfully describes the stress-strain behavior of the FRP-confined FLWAC with good agreement because the effect of fine aggregate on the ultimate strain was carefully considered. The proposed model also can be embedded in the numerical analysis for driving the compressive behavior of elements, and then analyze the global response of FRP-strengthened FLWAC structures.

## 7. Conclusions

The behavior of CFRP-confined FLWAC subjected to an axial compressive load was experimentally studied in this work. Test results of FLWAC were analyzed together with the test results of CFRP-confined LWAC and a related NC model. The strength capacity and ductility of FLWAC were significantly enhanced by CFRP jacketing. Using lightweight fine aggregates instead of normal weight fine aggregate can effectively reduce the self-weight of concrete but it does not lead to any reduction of strength. Nevertheless, the enhancement in ductility for the CFRP-confined FLWAC is distinctly larger than that obtained for the CFRP-confined LWAC and NC due to the lower density of lightweight fine aggregates. This discrepancy becomes more pronounced with the increase in FRP rigidity ratio. The dilation behavior of FRP-confined lightweight is found to be highly related to the aggregate type, and this effect further leads to different confinement mechanism caused by FRP confinement. These test results can further conclude that the coarse aggregate governs the strength properties of FRP-confined concrete, and the fine aggregate mainly controls its ductility properties. However, the internal damages condition of confined concrete and the interlock behavior between different aggregates are still unclear, which will be further studied in future using more advanced sensor technology, such as piezoceramic based smart aggregates.

Furthermore, the model that was developed by Zhou et al. [2] for CFRP-confined LWAC underestimates the ultimate strain of CFRP-confined FLWAC, especially for FLWAC specimens confined with three layers of CFRP. Hence, a new ultimate strain model was proposed in this study, which determines the effect of different aggregate types. The stress-strain models that were developed for FRP-confined LWAC columns are not capable of FRP-confined FLWAC columns unless the proposed ultimate strain model is used. By reasonably adopting the suitable ultimate strain model, the improved stress-strain model can predict the stress-strain behavior of FRP-confined FLWAC with good performance.

## Figures and Tables

**Table 1:** Summary of Specimen details and test results.

Specimen NO.	*f*_co_ (MPa)	*f_com_* (MPa)	*f_cc_* (MPa)	*FRP* Layers	*ε_co_* (%)	*ε_com_* (%)	*ε_cu_* (%)	*ε_hoop_* (%)	*k_s_*
New test data									
C40F0-1	37.1	39.8	/	0	0.188	0.151	/	/	/
C40F0-2	40.9		/	0	0.132		/	/	/
C40F0-3	41.4		/	0	0.136		/	/	/
C40F1-1	/	/	51.0	1	/	/	0.903	0.566	0.460
C40F1-2	/		52.7	1	/		0.632^a^	0.502	0.408 ^a^
C40F1-3	/		57.0	1	/		0.985	0.474	0.385 ^a^
C40F1-4	/		58.6	1	/		0.872	0.561	0.456
C40F1-5	/		54.4	1	/		0.919	0.571	0.464
C40F3-1	/	/	81.4	3	/	/	2.058	0.648	0.527
C40F3-2	/		83.2	3	/		2.384	0.899	0.730
C40F3-3	/		79.2	3	/		2.047	0.732	0.595
C40F3-4	/		93.0	3	/		2.616	0.572	0.465
C40F3-5	/		86.8	3	/		2.207	0.600	0.488
Zhou et al. [2]									
C1-1(1)	21.2		34.5	1	0.151		0.780	0.717	0.463
C1-1(2)			38.2	1			0.875	0.418	0.270 ^a^
C1-1(3)			49.9	1			1.003	0.598	0.398
C1-3(1)			54.6	3			3.389	0.701	0.453
C1-3(2)			52.8	3			3.475	0.769	0.496
C1-3(3)			54.5	3			3.641	0.787	0.508
C2-1(1)			57.2	1			0.708	0.756	0.488
C2-1(2)	38.8		56.2	1	0.180		0.631	0.814	0.526
C2-1(3)			56.7	1			3.332 ^a^	0.655	0.439 ^a^
C2-3(1)			85.5	3			1.506	0.805	0.520
C2-3(2)			87.1	3			1.559	0.814	0.526
C2-3(3)			83.4	3			1.516	0.885	0.571

*f_com_* and *ε_com_* are the mean compressive strength of plain concrete and its corresponding strain, respectively. Values denoted by “a” were deemed invalid due to their large deviation, and not considered for the analysis.

**Table 2:** Mix proportion and mechanical properties of full lightweight aggregate concrete (FLWAC).

Water kg/m^3^	Cement kg/m^3^	Coarse Aggregate kg/m^3^	Sand kg/m^3^	Silica Fume kg/m^3^	Water to Cement Ratio	Dry Density kg/m^3^	*f_co_* MPa	*ε_co_*_%_	*E_c_* GPa
150	450	477	405	50	0.30	1581	39.8	0.151	22.0

Note: *f_co_* = cylinder strength of FLWAC, *ε_co_* = axial peak strain of FLWAC, *E_c_* = elastic modulus of FLWAC.

**Table 3:** The main properties of lightweight coarse aggregates (LCAs).

Coarse Aggregate Type	24 h Water Absorption (%)	Bulk Density (kg/m^3^)	Apparent Density (kg/m^3^)	Tube Crushing Strength (MPa)
Class 800 crushed shale ceramsite	6.0	750	1272	6.4

**Table 4:** Aggregate grading results of LCAs.

Nominal Grain Size/mm	19	16	9.5	4.75	2.36	<2.36
screen residue (g)	133.3	730.4	3215.6	3552.9	353.7	167.0
cumulative screen residue (%)	1.6	10.6	50.1	93.6	97.7	100

**Table 5:** Aggregate grading results of lightweight fine aggregates (LFAs).

Nominal Grain Size/mm	2.5	1.25	0.63	0.315	0.16	<0.16
screen residue (g)	8.8	558.8	159.5	48.8	5.0	21.8
cumulative screen residue (%)	1.1	70.8	90.6	96.7	97.3	100
